# Assessment of atherosclerosis and endothelial dysfunction risk factors in patients with primary glomerulonephritis

**DOI:** 10.1590/2175-8239-JBN-2022-0116en

**Published:** 2023-01-13

**Authors:** Rodrigo Hagemann, Marcela Tatiana Watanabe, João Carlos Hueb, Luis Cuadrado Martín, Vanessa dos Santos Silva, Jacqueline do Socorro Costa Teixeira Caramori

**Affiliations:** 1 Universidade Estadual Paulista Júlio de Mesquita Filho, Faculdade de Medicina de Botucatu, Departamento de Clínica Médica, Botucatu, São Paulo, Brazil.; 2 Universidade Federal do Paraná, Complexo Hospital de Clínicas, Departamento de Clínica Médica, Curitiba, Paraná, Brazil.

**Keywords:** Glomerulonephritis, Renal Insufficiency, Chronic, Chronic Kidney Disease-Mineral and Bone Disorder, Cardiovascular diseases, Glomerulonefrite, Insuficiência Renal Crônica, Distúrbio Mineral e Ósseo na Doença Renal Crônica, Doenças Cardiovasculares

## Abstract

**Introduction:**

Glomerulonephritis are the third cause of chronic kidney disease (CKD) requiring dialysis in Brazil. Mineral and bone disorder (MBD) is one of the complications of CKD and is already present in the early stages. Assessment of carotid intima-media thickness (CIMT) and flow-mediated vasodilatation (FMV) are non-invasive ways of assessing cardiovascular risk.

**Hypothesis:**

Patients with primary glomerulonephritis (PG) have high prevalence of atherosclerosis and endothelial dysfunction, not fully explained by traditional risk factors, but probably influenced by the early onset of MBD.

**Objective:**

To evaluate the main markers of atherosclerosis in patients with PG.

**Method:**

Clinical, observational, cross-sectional and controlled study. Patients with PG were included and those under 18 years of age, pregnants, those with less than three months of follow-up and those with secondary glomerulonephritis were excluded. Those who, at the time of exams collection, had proteinuria higher than 6 grams/24 hours and using prednisone at doses higher than 0.2 mg/kg/day were also excluded.

**Results:**

95 patients were included, 88 collected the exams, 1 was excluded and 23 did not undergo the ultrasound scan. Patients with PG had a higher mean CIMT compared to controls (0.66 versus 0.60), p = 0.003. After multivariate analysis, age and values for systolic blood pressure (SBP), FMV and GFR (p = 0.02); and FMV and serum uric acid (p = 0.048) remained statistically relevant.

**Discussion and conclusion:**

The higher cardiovascular risk in patients with PG was not explained by early MBD. Randomized and multicentric clinical studies are necessary to better assess this hypothesis.

## Resumo

**Introdução:**

Glomerulopatias são a terceira causa de doença renal crônica (DRC) com necessidade de diálise no Brasil. Distúrbio mineral e ósseo (DMO) é uma das complicações da DRC e está presente já nos estágios iniciais. A avaliação da espessura médio-intimal de carótidas (EMIC) e da vasodilatação fluxo-mediada (VFM) são maneiras não invasivas de avaliação do risco cardiovascular.

**Hipótese:**

Pacientes com glomerulopatias primárias (GP) apresentam alta prevalência de aterosclerose e disfunção endotelial, não explicada totalmente pelos fatores de risco tradicionais, mas provavelmente influenciada pela instalação precoce do DMO.

**Objetivo:**

Avaliar os principais marcadores de aterosclerose em pacientes com GP.

**Método:**

Estudo clínico, observacional, transversal e controlado. Foram incluídos portadores de GP e excluídos menores de 18 anos, gestantes, menos de três meses de seguimento e os com glomerulopatia secundária. Também foram excluídos aqueles que, no momento da coleta, apresentavam proteinúria maior que 6 gramas/24 horas e uso de prednisona em doses superiores a 0,2 mg/kg/dia.

**Resultados:**

95 pacientes foram incluídos, 88 colheram os exames, 1 foi excluído e 23 não realizaram a ultrassonografia. Os pacientes com GP apresentaram maior EMIC média em relação ao controle (0,66 *versus* 0,60), p = 0,003. Após análise multivariada, mantiveram relevância estatística a idade e os valores de pressão arterial sistólica (PAS), VFM e TFG (p = 0,02) e VFM e ácido úrico sérico (p = 0,048).

**Discussão e conclusão:**

Pacientes com GP apresentaram maior risco cardiovascular, entretanto esse risco não foi explicitado pelo DMO precoce. Estudos clínicos randomizados e multicêntricos são necessários para melhor determinação dessa hipótese.

## Introduction

Glomerulonephritis have different forms of clinical and laboratory presentation. They may manifest as nephritic or nephrotic syndromes, isolated microscopic hematuria, macroscopic hematuria, isolated proteinuria or, in some rare cases, even with rapid loss of kidney function^1^. They can be classified as secondary, when resulting from systemic diseases, such as systemic lupus erythematosus (SLE), or primary, when originating in the kidney itself. The main histological patterns of primary glomerulonephritis are: immunoglobulin A nephropathy (IgAN), minimal change disease (MCD), focal segmental glomerulosclerosis (FSGS), membranous nephropathy (MN) and membranoproliferative glomerulonephritis.

According to the Census from the Brazilian Society of Nephrology, glomerulonephritis are the third underlying disease that most leads patients to need renal replacement therapy^2^.

One of the complications of chronic kidney disease (CKD) is the development of mineral and bone disorders (MBD)^3^. Hyperphosphatemia is one of the main promoters of vascular calcification in patients with CKD in more advanced stages, and it is associated with higher mortality in these patients^4^.

The association of high levels of PTH, calcium, phosphate and increased mortality in hemodialysis patients is already well documented in the literature^5^. Recent studies also show this association in patients with different stages of CKD and even in individuals with normal kidney function^6,7^.

The fact that even individuals with normal levels of calcium, phosphate and PTH may present increased vascular calcification and mortality led to the search for early MBD markers. Japanese researchers, in the year 2000, identified a new factor from the family of fibroblast growth factors, called FGF-23^8^. Bone tissue, due to the high levels of FGF-23 expression by osteocytes, is considered the main source of its production.

In addition to elevated FGF-23 levels, patients with CKD in more advanced stages have low serum levels of soluble klotho. This deficiency is associated with vascular calcification, cardiac fibrosis and ventricular hypertrophy^9^.

The process of atheromatous plaques formation has been studied for a long time and is considered a chronic inflammatory process, a response of the endothelium to a series of aggressors, such as hypertension, diabetes mellitus (DM), smoking, obesity and dyslipidemia^10^. These aggressors are known as traditional risk factors and have already been widely evaluated in prospective studies with large numbers of participants, such as the Framingham study, carried out in the United States of America^11^.

More recently, other risk factors for atherosclerosis, called non-traditional risk factors, have been pointed out, since the traditional ones alone do not fully explain the increased cardiovascular risk in patients with CKD^12^ nor in patients with primary glomerulonephritis^13^.

Nephrotic syndrome, associated with an adverse lipid profile and increased risk of thrombotic events, increases the risk of cardiovascular disease (CVD)^14^. Isolated proteinuria, even without the other components of nephrotic syndrome, is already a well-defined cardiovascular risk marker^15^ and, in addition to it, CKD itself is a risk factor for adverse cardiovascular events^16^.

Chronic inflammatory diseases, such as some autoimmune rheumatic diseases, favor the atherosclerotic process, and the most common cause of death in patients with these diseases is linked to adverse cardiovascular events^17^. Thus, inflammation is a non-traditional risk factor for the development of atherosclerosis.

FGF-23 seems to be involved in chronic inflammation, as its formation is stimulated by the pro-inflammatory transcription factor NF-kB and by other cytokines^18^.

Cozzolino et al.^19^ postulated a hypothesis linking two traditional factors of CKD progression, high blood pressure and proteinuria, to phosphate metabolism. According to these authors, the FGF-23/klotho system is strongly connected to the renin-angiotensin-aldosterone system, and high serum phosphate levels may reduce the nephroprotective effect of inhibitors of this system.

Arteriography is the gold standard test for diagnosing atherosclerosis, but it is invasive and poses risks to the patient. On the other hand, carotid intima-media thickness (CIMT) is a valid marker with good predictive value for adverse cardiovascular events in the general population^20^ and in patients with CKD^21^. The increase in CIMT occurs before the formation of atheromatous plaque, and individuals with a faster increase in CIMT have a higher cardiovascular risk^22^. CIMT values greater than 0.9 mm are strong predictors of adverse cardiovascular events^23^.

In addition to CIMT analysis, another way to assess endothelial dysfunction is through flow-mediated vasodilatation (FMV)^24^. Individuals with FMV values below 10% have an increased cardiovascular risk. Dogra et al.^25^ evaluated 38 patients (19 with nephrotic syndrome and 19 in the control group) and reported that those with nephrotic syndrome had lower MFV values, with statistical significance (p = 0.02).

To date, there is a lack of investigations relating early mineral and bone disorders with the development of atherosclerosis and endothelial dysfunction in patients with primary glomerulonephritis.

Patients with primary glomerulonephritis have a high prevalence of atherosclerosis and endothelial dysfunction, not fully explained by traditional risk factors, but possibly influenced by the early onset of mineral and bone disorders, marked by increased serum levels of FGF-23 and chronic inflammation.

The objective of the present study was to evaluate the main markers of atherosclerosis in patients with primary glomerulonephritis, including non-traditional risk factors, in addition to comparing CIMT values between patients with primary glomerulonephritis and healthy volunteers, and to investigate which cardiovascular risk factors are associated with higher CIMT and worse FMV.

## Methods

Clinical, observational, cross-sectional and controlled study, with quantitative and statistical evaluation of clinical, laboratory and ultrasound data.

This study was carried out at the Glomerulonephritis Outpatient of the Botucatu Medical School. 

All patients followed at the Outpatient Glomerulonephritis, with primary glomerulonephritis, documented by renal biopsy, were invited to participate of the study. Patients under 18 years of age, pregnants, those under follow-up for less than three months, and those with secondary glomerulonephritis were excluded. Those who, at the time of inclusion, had proteinuria higher than 6 grams in 24 hours and using prednisone at doses higher than 0.2 mg/kg/day in the last three months were also excluded.

The control group consisted of volunteers who participated in a previous study, when they were selected among blood donors from the Botucatu Medical School signed the Informed Consent Form, underwent clinical evaluation, collection of laboratory tests and ultrasonographic evaluation^26^. It is noteworthy that the CIMT assessment was performed by the same examiner and with the same device used in the present study. It is, therefore, a historical control group^27^.

Only patients who, after initial contact, expressed a desire to participate in the study voluntarily and who signed the Free and Informed Consent Term participated in the study. This study was approved by the Research Ethics Committee of the Medical School of Botucatu – Unesp.

The patients were evaluated at three different moments: clinical evaluation, nutritional evaluation with collection of exams and ultrasound evaluation.

The medical interview was carried out by the researcher himself, who collected data regarding treatment duration, the underlying disease, medication used, total corticosteroid load, total load of calcium received and vitamin D supplementation. They were also investigated regarding the history of coronary artery disease (CAD), cerebrovascular event and peripheral vascular disease. For the present study, the occurrence of AMI, coronary events confirmed by cardiac catheterization and surgical revascularization were considered CAD. Cerebral vascular events were both ischemic and hemorrhagic. Peripheral vascular disease was defined as the need for treatment with angioplasty for lower limb ischemia, lower limb amputation, and intermittent claudication. To measure the systemic blood pressure, measurements were taken in both arms a few minutes after the patient remained seated. If there was a discrepancy greater than 4 mmHg between the values, a third measurement was performed on the arm with the highest blood pressure value, and the recorded value was an average of these measurements.

In a second moment, the patients attended, by appointment, to the Clinical Research Unit of Botucatu Medical School (Upeclin), where they realized the collection of laboratory tests and a nutritional assessment. The glomerular filtration rate was estimated using the CKD-EPI^28^ equation. The method used to measure PTH was chemiluminescence, and the unit used was pg/mL. Two tubes were separated, centrifuged and stored. For vitamin D analysis, 25(OH)D3 was measured using the HPLC technique (high performance liquid chromatography). FGF-23 dosages were performed at the end of the collection period. The technique used was ELISA (Enzyme-Linked Immunosorbent Assay) sandwich, and the dosages of the intact molecule were performed in duplicate, and then the averages of the measured values, expressed in pg/mL, were recorded. Nutritional assessment was performed by an experienced nutritionist from the Medical School of Botucatu.

Ultrasound examinations were performed by a single experienced and duly qualified examiner. CIMT was obtained by an automated method, establishing maximum and average thickness. The images were obtained and analyzed following the recommendations of the Consensus Statement from the American Society of Echocardiography^29^ and the Mannheim Carotid Intima-Media Thickness Consensus^30^.

To assess the FMV, the tests were performed according to the recommendations from the Guidelines for the Ultrasound Assessment of Endothelial-Dependent Flow-Mediated Vasodilatation of the Brachial Artery^31^. FMV values lower than 10% are indicative of endothelial dysfunction.

### Data Analysis Procedure

We used the Fisher and Belle's formula^32^ to calculate the sample size with the following variables: prevalence of atherosclerosis in patients with SLE and GFR higher than 30 mL/min/1.73m^2^ (around 40%)^33^, interval of 95% confidence and 10% sampling error. The result was 96 patients.

Categorical variables were compared using the chi-square test or Fisher's exact test, when appropriate. Continuous variables and those with parametric distribution were compared using the t-test for independent samples, and those with non-parametric distribution using the Mann-Whitney test.

To verify the distribution of each variable, normality tests were performed using the Kolmogorov-Smirnov and Shapiro-Wilk tests.

The data were expressed as mean ± standard deviation, median, first and third quartiles or percentage, when appropriate. A value of p < 0.05 was considered statistically significant.

The variables that differed at the 10% level were submitted to a multivariate analysis. Due to the fact that most of them have non-parametric distribution, we used a generalized linear model^34^. This type of model is an extension of the simple and multiple regression models and enables the use of other distributions for the errors and a link function relating the mean of the response variable to the linear combination of the explanatory variables. With these generalized linear models, it is possible to model variables of interest that take the form of counting, continuous symmetric and asymmetric, binary and categorical. Gamma regression was used, which models positive and asymmetric data.

Pearson correlation was used for parametric distribution variables and Spearman correlation was used for non-parametric variables.

The analysis of the data from each participant was carried out in the SPSS 22 program.

## Results

From March 2016 to November 2017, 378 patients were seen at the Glomerulonephritis Outpatient of Botucatu Medical School. Of these, 134 met the inclusion criteria for the study and 95 agreed to participate. Of this group, 88 collected laboratory tests and 64 were also submitted to ultrasound evaluation. Among those who did not complete the evaluation, 23 did not undergo the ultrasound examination and one patient was excluded because he had a proteinuria of 10.51 grams/24 hours at the time of collection.

The most frequent glomerulonephritis in the group of 64 patients who completed the assessment was podocytopathy, represented by MCD/FSGS, with 32 patients (50% of cases). Membranous nephropathy was the second most frequent, with 19 patients (30%), and IgAN the third (20%), with 13 patients.

The clinical and laboratory characteristics of the 64 patients who completed the assessment and the 23 who did not undergo ultrasound examinations were similar.

Table 1 shows the MBD profile according to the primary glomerulonephritis. No statistically significant differences were observed between the histological type and the evaluated parameters.

**Table 1:** Characteristics of patients according to their glomerulonephritis

	Age	PTH	Vitamin D	FGF-23	CIMT	FMV
MCD/FSGS n = 32	45.39 ± 15.51	26.4 (15.6; 40.7)	40.51 ± 11.38	238.7 (63.3; 488.6)	0.66 ± 0.13 N = 32	8.2 (5.5;12.9) N = 32
MN n = 19	50.6 ± 15.11	13.3 (7.6; 21.2)	39.06 ± 11	282.8 (77; 655.6)	0.68 ± 0.14 N = 19	11.1(6.6;14.8) N = 19
IgAN n = 13	44.3 ± 10.59	33.45 (29.72; 50.75)	43.69 ± 9.32	157.2 (39.2; 564.8)	0.61 ± 0.08 N = 13	8.8 (6.7;13.8) N = 13

PTH: parathormone; FGF-23: fibroblast growth factor 23; CIMT: carotid intima-media thickness; FMV: flow-mediated vasodilatation; MCD: minimal change disease; FSGS: focal and segmental glomerulosclerosis; MN: membranous nephropathy; IgAN: IgA nephropathy.

Characteristics of participants in the control group and patients with glomerulonephritis are shown in Table 2. We emphasize that patients with glomerulonephritis had higher mean CIMT values (0.66 ± 0.12 versus 0.60 ± 0.12, p = 0. 03).

**Table 2:** Clinical and laboratory data of the groups

	Control group (n = 70)	Glomerulonephritis group (n = 64)	p
Age (years)	42.03 ± 11.07	45.61 ± 15.26	0.13
Female (n,%)	48 (68.58)	38 (59.38)	0.27
Caucasian (n,%)	57 (81.43)	56 (87.5)	0.163
BMI (kg/m^2^)	26.59 (24.35; 29.70)	28.76 (25.29; 31.40)	0.07
Smokers (n,%)	9 (12.86)	19 (29.68)	0.33
Hypertension (n,%)	10 (14.29)	50 (78.12)	<0.01
DM (n,%)	6 (8.57)	11 (17.19)	0.13
Statins use (n,%)	6 (8.57)	38 (59.37)	<0.01
SBP (mmHg)	120 (110;130)	130 (118;142)	<0.01
DBP (mmHg)	80 (70;80)	82 (73.50; 90)	0.01
Creatinine (mg/dL)	0.80 (0.70;1)	0.95 (0.80; 1.32)	<0.01
eGFR (mL/min/1,73 m^2^)	98.70 (85.62; 111.37)	80.30 (48; 105.12)	<0.01
Urea (mg/dL)	30 (23.50; 34.80)	40 (31.25; 52.50)	<0.01
Glucose (mg/dL)	86 (79.25; 97.50)	85 (79.75;91.25)	0.52
Total cholesterol (mg/dL)	189.54 ± 33.66	178.77 ± 41.25	0.10
HDL cholesterol (mg/dL)	51 ± 13.29	48.65 ± 11.74	0.28
LDL cholesterol (mg/dL)	110.90 ± 30.12	101.68 ± 36.68	0.12
Triglycerides (mg/dL)	109 (77.25; 169.50)	114.50 (77;182)	0.69
CIMT (mm)	0.60 ± 0.12	0.66 ± 0.12	0.003

BMI: body mass index; DM: diabetes mellitus; SBP: systolic blood pressure; DBP: diastolic blood pressure; eGFR: estimated glomerular filtration rate; HDL: high density lipoproteins; LDL: low density lipoproteins; CIMT: carotid intima-media thickness.

As most of the variables presented had non-parametric distribution, a generalized linear model was used, with gamma regression for analysis (Table 3). The variables that maintained statistical relevance were age and systolic blood pressure.

**Table 3:** Gama regression for CIMT

Parameter	β	95% confidence interval		P
		Inferior	Superior	
Age	0.004	0.002	0.007	0.001
Systolic blood pressure	0.003	0.001	0.005	0.001
Statins	–0.056	–0.129	0.018	0.140
eGFR	<0.001	–0.001	0.002	0.707
Body mass index	<0.001	–0.004	0.005	0.851

CIMT: carotid intima media thickness; eGFR: estimated glomerular filtration rate.

The 64 patients who completed the assessment were divided into four quartiles according to CIMT values. Values below 0.575 mm constituted the first quartile, with 15 patients; values between 0.576 and 0.65 constituted the second quartile, with 17 patients; values between 0.651 and 0.734 formed the third quartile, with 16 patients; and values higher than 0.734 formed the fourth quartile, with 16 patients.

For this analysis, two groups were considered: the first including cases with CIMT < 0.734 (first three quartiles) and the second including cases with CIMT ≥ 0.734 (fourth quartile). The groups were compared regarding age, FGF23, proteinuria, GFR, flow-mediated dilation and endothelial dysfunction.

For variables that met the condition of normality (age, Log FGF23 and estimated GFR), we used the Student's t test. For variables that did not meet the condition of normality (proteinuria, FGF23 and mediated flow dilation), the Mann-Whitney test was used (Table 4). Patients with CIMT ≥ 0.734 were associated with older age (p < 0.01) and lower flow-mediated vasodilatation (0.03), as shown in Table 4.

**Table 4:** Comparison between the first three and the fourth quartiles of CIMT

Variable	Group	n	Mean	Standard deviation	Median	Minimum	Maximum	IQR	p
Age (years)*	<0.734 ≥0.734	48 16	41.0 59.5	12.7 14.0	41 59.5	18 25	78 86	19 18	<0.001
Proteinuria**	<0.734 ≥0.734	48 16	1.09 0.86	1.13 1.09	0.64 0.37	0.05 0.09	4.68 3.98	1.26 1.08	0.264
FGF23**	<0.734 ≥0.734	47 16	2783 869	10872 1302	211 321	6.35 39.6	68780 5044	443 998	0.201
Log FGF23*	<0.734 ≥0.734	47 16	5.41 5.87	1.89 1.43	5.35 5.77	1.85 3.68	11.1 8.53	2.04 2.66	0.373
Flow-mediated vasodilatation**	<0.734 ≥0.734	48 16	12.8 8.35	8.98 5.99	9.45 6.45	3.77 1.96	40.7 23.3	8.02 7.77	0.033

IQR: interquartile range.

* Student t test for parametric variables.

** Mann-Whitney test for non-parametric variables.

Normal distribution variables were evaluated using the Pearson's correlation, with the following results: age with mean CIMT (r = 0.607; p < 0.001) and vitamin D with mean CIMT (r = 0.218; p = 0.091).

For non-parametric distribution variables, we used Spearman's correlation, and the values are shown in Table 5, highlighting: SBP with mean CIMT (r = 0.388; p = 0.002), BMI with FMV (r = –0.262; p = 0.036), mean GFR with CIMT (r = –0.247; p = 0.049) and with FMV (r = 0.317; p = 0.011), duration of hypertension with FMV (r = –0.262; p = 0.036), serum uric acid levels and FMV (r = –0.347; p = 0.005), glycemia and mean CIMT (r = 0.382; p = 0.002) and triglycerides and FMV (r = –0.425; p < 0.001).

**Table 5:** Clinical variables and outcomes correlations

		CIMT	FMV
FGF-23	r	0,126	0,057
	p	0,325	0,655
Vitamin D	r	0,218*	0,034
	p	0,091*	0,791
Age	r	0,607*	–0,139
	p	<0,01*	0,274
SBP	r	0,388	–0,126
	p	0,002	0,320
BMI	r	0,212	–0,262
	p	0,092	0,036
eGFR	r	–0,247	0,317
	p	0,049	0,011
Proteinuria	r	–0,218	–0,007
	p	0,084	0,954
Follow-up duration	r	0,312	–0,182
	p	0,012	0,150
Hypertension duration	r	0,133	–0,262
	p	0,293	0,036
Uric acid	r	0,003*	–0,347
	p	0,980*	0,005
Glucose	r	0,382	–0,137
	p	0,002	0,281
Triglycerides	r	0,116	–0,425
	p	0,362	<0,001
Phosphate intake	r	0,013	–0,224
	p	0,923	0,096

CIMT: carotid intima media thickness; FMV: flow-mediated vasodilatation; FGF-23: fibroblast growth factor 23; SBP: systolic blood pressure; BMI: body mass index; eGFR: estimated glomerular filtration rate.

* Pearson correlation; the others variables were analyzed by Spearman correlation.

For the multivariate analysis of these correlations, we used a gamma regression model, and only the variable age maintained a statistically significant correlation with the average CIMT measurements (p < 0.01). Regarding FMV, only the glomerular filtration rate (p = 0.02) and serum uric acid levels (p = 0.048) remained statistically relevant.

## Discussion

The present study showed that patients with primary glomerulonephritis had a higher cardiovascular risk compared to the general population, marked by a higher mean CIMT. However, this increased risk was not associated with the markers investigated, which were still little altered by the early onset of the mineral and bone disorder, not providing the expected support for the hypothesis of this study.

Patients with primary glomerulonephritiss had higher mean CIMT compared to the control group, indicating a higher cardiovascular risk in this population^20^. It is still unclear whether this increased risk is attributable to glomerular disease itself or to the concomitant presence of other cardiovascular risk factors and CKD.

To assess this issue, Hutton et al.^35^ conducted an observational study of a Canadian cohort of 2,544 patients with CKD (GFR between 15 and 45 ml/min/1.73m^2^) followed for three years. The patients were divided into two groups: with glomerulonephritis (excluding those undergoing immunosuppressive treatment during the period) and those with CKD secondary to other etiologies. There was matching for age, sex, race, presence of DM, previous cardiovascular event, GFR, SBP, statin use and lipid profile, resulting in 272 in each group. The primary outcome was the occurrence of a cardiovascular event, defined as fatal and non-fatal AMI, myocardial revascularization, ischemic stroke or onset of congestive heart failure. Cardiovascular risk was similar between the two groups, suggesting a greater influence of previous risk factors and low GFR than the etiology of CKD.

In the present study, the number of patients with DM was equivalent in both groups, which also did not differ in terms of sex, race, smoking and lipid profile. In the glomerulonephritis group, there was a higher prevalence of hypertensive and CKD patients. After multivariate analysis, only age and SBP values remained statistically relevant in relation to CIMT. When the analyzed parameter was FMV, in the group of patients with glomerulonephritis, there was an inverse correlation with GFR and serum uric acid levels.

In this cross-sectional study, we were able to show the relevance of GFR and traditional risk factors, confirming the findings described by Hutton et al.^35^.

Since glomerulonephritis itself was not associated with increased cardiovascular risk, by excluding patients with nephrotic proteinuria and consequently greater disease activity, it is necessary to identify and treat other risk factors, such as high uric acid levels.

In the present study, we found that the higher uric acid levels, the lower the FMV, what increases the cardiovascular risk in patients with primary glomerulonephritis.

The increase in uric acid levels inside the cells is associated with a reduction in nitric oxide metabolites, what may explain the endothelial dysfunction seen in these patients^36^. It is noteworthy that there is still a relationship between high uric acid levels and the renin-angiotensin-aldosterone system activation^37^.

Regarding patients with primary glomerulonephritis, there is a study with 353 patients with IgA nephropathy^38^, with a mean follow-up time of five years; in the pilot study phase, 40 patients with IgAN and hyperuricemia were recruited and the effect of uric acid reduction with allopurinol on the preservation of kidney function and the use of antihypertensive drugs was evaluated. The allopurinol group comprised 21 patients and the control group comprised the other 19. None of the patients used ACE inhibitors/ARBs. Analyzes of the retrospective study showed that hyperuricemia is a risk factor for CKD progression in patients with IgAN, regardless of GFR, unlike what is found among the general causes of CKD. The clinical study did not show the benefit of reducing uric acid level in the evolution of CKD or proteinuria, but it did show benefits in controlling systemic blood pressure, with a reduction in the antihypertensive drugs dose in the allopurinol group. A decrease in GFR in the first month of treatment with allopurinol was also reported, similar to the hemodynamic effect found at the beginning of treatment with ACEI/ARB, reinforcing the association of high levels of uric acid with RAAS activation.

It is indisputable that there is a lack of clinical and randomized studies to assess the influence of serum uric acid levels in patients with primary glomerulonephritis.

The hypothesis that early MBD, marked by increased plasma concentrations of FGF-23, would influence the atherosclerosis process in patients with primary glomerulonephritis was not confirmed in our evaluation, which was cross-sectional.

Possible explanations could be the heterogeneity of measured FGF-23 values, a fact already reported in other studies^39^, the diversity of laboratory kits and the fact that some studies, such as the present one, measure the intact molecule while others only measure the active portion. Another important issue would be the outcome assessed. There is evidence of an association between elevated FGF-23 values and left ventricular hypertrophy^40^, but little evidence of an association with CIMT and FMV.

The present study was cross-sectional and carried out in a single center, and had a historical control group. The number of patients required was not reached, so there is no way to exclude the occurrence of a type II error. This reinforces the importance of carrying out multicenter studies in the future, in order to recruit a larger number of patients. As previously discussed, the type of FGF-23 measurement performed and the chosen clinical outcomes may have an influence. It is important to mention the lack of data regarding the time each patient was exposed to proteinuria, important information that should be included in future studies on this subject.

Other limitations of the present study were the heterogeneity of the sample, as patients with different stages of CKD and with different primary glomerulonephritis were included, and also the absence of the total amount of corticosteroids used by patients over time. It should also be taken into account that diseases with a more nephrotic pattern, such as MCD, FSGS and NM, may have different outcomes from those with a more nephritic pattern, such as IgAN. The small number of patients in the present study showed no difference, but we plan to perform an outcome analysis of the present sample after a period of 10 years, in order to verify whether there was a difference in the outcome between these groups.

However, this is a pioneering study on some aspects. Few studies have evaluated bone mineral disorders and glomerular diseases. Dias et al.^41^ showed that patients with primary glomerulonephritis had low bone formation and high resorption, in addition to low osteoblast proliferation. The present study concluded that patients with primary glomerulonephritis form a group with heterogeneous characteristics exposed to several cardiovascular risk factors. In our study, there was an association between increased cardiovascular risk and high levels of SBP and serum uric acid levels; however, randomized clinical and intervention studies are needed to assess the benefits of a strict control of systemic blood pressure and control of serum uric acid levels in patients with primary glomerulonephritis.
